# Improving Randomized-Controlled Trials in Foot and Ankle Orthopaedics: The Need to Include Sociodemographic Patient Data

**DOI:** 10.1177/19386400231170965

**Published:** 2023-05-06

**Authors:** Brandon J. Martinazzi, Gregory J. Kirchner, Hannah H. Nam, Kirsten Mansfield, Kelly Dopke, Anna Ptasinski, Adeshina Adeyemo, Kempland C. Walley, Michael C. Aynardi

**Affiliations:** Penn State College of Medicine, Hershey, Pennsylvania; Department of Orthopaedics and Rehabilitation, Milton S. Hershey Medical Center, Penn State College of Medicine, Hershey, Pennsylvania; Penn State College of Medicine, Hershey, Pennsylvania; Penn State College of Medicine, Hershey, Pennsylvania; Penn State College of Medicine, Hershey, Pennsylvania; Penn State College of Medicine, Hershey, Pennsylvania; Department of Orthopaedics and Rehabilitation, Milton S. Hershey Medical Center, Penn State College of Medicine, Hershey, Pennsylvania; Department of Orthopaedic Surgery, University of Michigan, Ann Arbor, Michigan; Penn State College of Medicine, Hershey, Pennsylvania; Department of Orthopaedics and Rehabilitation, Milton S. Hershey Medical Center, Penn State College of Medicine, Hershey, Pennsylvania

**Keywords:** sociodemographic data, randomized-controlled trials, foot and ankle surgery

## Abstract

**Background:**

The representation of sociodemographic data within randomized-controlled trials (RCT) regarding foot and ankle surgery is undefined. The purpose of this study was to determine the incidence of sociodemographic data being reported in contemporary foot and ankle RCTs.

**Methods:**

Randomized-controlled trials within the PubMed database from 2016 to 2021 were searched and the full text of 40 articles was reviewed to identify sociodemographic variables reported in the manuscript. Data regarding race, ethnicity, insurance status, income, work status, and education were collected.

**Results:**

Race was reported in the results in 4 studies (10.0%), ethnicity in 1 (2.5%), insurance status in 0 (0%), income in 1 (2.5%), work status in 3 (7.5%) and education in 2 (5.0%). In any section other than the results, race was reported in 6 studies (15.0%), ethnicity in 1 (2.5%), insurance status in 3 (7.5%), income in 6 (15.0%), work status in 6 (15.0%), and education in 3 (7.5%). There was no difference in sociodemographic data by journal (P = .212), year of publication (P = .216), or outcome study (P = .604).

**Conclusion:**

The overall rate of sociodemographic data reported in foot and ankle RCTs is low. There was no difference in the reporting of sociodemographic data between journal, year of publication, or outcome study.

**Level of Evidence:**

Level II

“Our study demonstrates that there is a significant lack of sociodemographic data reported across foot and ankle RCTs over the past 6 years.”

## Introduction

Social determinants of health, including but not limited to race, ethnicity, education, insurance coverage, income, and work status influence access to healthcare and drive population health disparities.^1-3^ With regards to orthopaedic surgery, social determinants of health are known to impact outcomes.^4-9^ Despite the important role these factors play, prior research has described a lack of diversity reported in orthopaedic clinical trials.^10^ As the population continues to diversify, reporting sociodemographic data in contemporary studies is becoming increasingly important.^11^

The population of patients treated by foot and ankle surgeons is no exception. For example, Singh and Cleveland found that age, race, ethnicity, and insurance status, were independently associated with total ankle arthroplasty outcomes.^12^ Furthermore, Bernstein et al suggested that income status should be considered when using Patient Acceptable Symptom State (PASS) to evaluate foot and ankle patient satisfaction following treatment.^13^ Other research has identified an association between race and ethnicity and the risk of amputation among Medicare patients with diabetic foot ulcers and infections.^14^

Randomized-controlled trials (RCTs) are prospective studies by nature, which allows researchers to select and include specific variables for analysis. Consequently, the representation of sociodemographic data included in RCTs can be used as a metric to understand the value of these variables within the foot and ankle community. To date, the literature is devoid of studies exploring the incidence of reported sociodemographic variables in RCTs. Therefore, the purpose of this study is to determine the proportion of contemporary foot and ankle related RCTs that report sociodemographic data.

## Materials and Methods

A literature review was conducted to identify RCTs related to foot and ankle surgery published within the last 6 years. The PubMed database was queried using the search term “Foot and Ankle Surgery” for RCTs published from 2016 to 2021 that were related to foot and ankle orthopedics. Each article’s journal of publication, year of publication, and outcome of interest was recorded. Articles were then separated into the following outcomes studies: infection prevention, pain control, surgical outcomes, and nonoperative outcomes. The full text of each article was reviewed to identify sociodemographic variables within the results or tables versus any other part of the article (i.e., results or otherwise). The sociodemographic variables that were collected include race, ethnicity, insurance status, income, work status, and education.

The primary outcome of this study was inclusion of sociodemographic variables within the results of RCTs, while a secondary outcome was inclusion of sociodemographic data in any other section besides the results of the RCT. Outcomes were reported as counts and percentages and a Fisher exact test was performed to determine the association between sociodemographic data included and independent variables (journal, year of publication, and outcome study of interest). For the purposes of our study, statistical significance was defined as *P* < 0.05.

## Results

From 2016 to 2021, 40 studies were identified across 17 different journals. RCTs were most commonly published in *Foot and Ankle Surgery* (15/40, 37.5%) and *Foot and Ankle International* (5/40, 12.5%). There were fewer RCTs in 2017 (2/40, 5.0%) compared with 2016 (5/40, 12.5%), 2018 (4/40, 10.0%), 2019 (8/40, 20.0%), 2020 (9/40, 22.5%), and 2021 (12/40, 30.0%). In our study, surgical outcomes represented the most common foot and ankle RCT (15/40, 37.5%), followed by pain control (14/40, 35.0%), nonoperative outcomes (7/40, 17.5%), and finally infection prevention (4/40, 10.0%). These results are reported in Table 1.

**Table 1.** Journal, Year of Publication, and Outcome Study of Randomized-Controlled Trials.

	*n* (%)
Journal	
*Foot and Ankle Surgery*	15 (37.5)
*Foot and Ankle International*	5 (12.5)
*Journal of Bone and Joint Surgery*	5 (12.5)
*International Orthopaedics*	2 (5.0)
Other	13 (32.5)
Year of publication	
2021	12 (30.0)
2020	9 (22.5)
2019	8 (20.0)
2018	4 (10.0)
2017	2 (5.0)
2016	5 (12.5)
Outcome study	
Surgical outcomes	15 (37.5)
Pain control	14 (35.0)
Nonoperative outcomes	7 (17.5)
Infection prevention	4 (10.0)

The inclusion of specific sociodemographic characteristics are reported in Table 2. The most commonly reported sociodemographic factor within the “Results” section was race (4/40, 10.0%), followed by work status (3/40, 7.5%), education (2/40, 5.0%), income (1/40, 2.5%), and finally ethnicity (1/40, 2.5%). Insurance status was not reported in any results within our sample (0/40, 0%). The most common sociodemographic factor reported in sections other than the results was race (6/40, 15.0%), income (6/40, 15.0%), work status (6/40, 15.0%), followed by insurance status (3/40, 7.5%) and education (3/40, 7.5%), and finally ethnicity (1/40, 2.5%).

**Table 2.** Sociodemographic Characteristics Included in Randomized-Controlled Trials.

	Included in results, *n* (%)	Included in any section, *n* (%)
Race	4 (10.0)	6 (15.0)
Ethnicity	1 (2.5)	1 (2.5)
Insurance status	0 (0.0)	3 (7.5)
Income	1 (2.5)	6 (15.0)
Work status	3 (7.5)	6 (15.0)
Education	2 (5.0)	3 (7.5)

As shown in Table 3, statistical comparison did not reveal any association between journal of publication and inclusion of sociodemographic factors (*P* = .212), year of publication and inclusion of sociodemographic factors (*P* = .216), or outcome study and inclusion of sociodemographic factors (*P* = .604).

**Table 3.** Comparison of Sociodemographic Data Included in Results of Randomized-Controlled Trials by Journal, Year of Publication, and Outcome Study.

	*n*/total	%	*P* value
Journal			.212
*Foot and Ankle Surgery*	1/15	6.7	
*Foot and Ankle International*	1/5	20.0	
*Journal of Bone and Joint Surgery*	2/5	40.0	
*International Orthopaedics*	1/2	50.0	
Other	1/13	7.7	
Year of publication			.216
2021	3/12	25.0	
2020	0/9	0.0	
2019	0/8	0.0	
2018	1/4	25.0	
2017	0/2	0	
2016	2/5	40.0	
Outcome Study			.604
Surgical outcomes	3/15	20.0	
Pain control	2/14	14.3	
Nonoperative outcomes	0/7	0.0	
Infection prevention	1/4	25.0	

## Discussion

Previous studies have criticized the lack of sociodemographic data included in RCTs in various medical and surgical disciplines.^15-17^ Numerous large-scale studies have demonstrated that social determinants, including race, ethnicity, insurance status, income, and education level influence patient experience, surgical outcomes, and postoperative complications.^7^ However, despite these relationships described in the literature, the majority of foot and ankle RCTs continue to lack sociodemographic factors in the study design and data analysis.^18^ To our knowledge, our study is the first to (1) evaluate which specific sociodemographic elements are most commonly reported in foot and ankle RCTs and (2) examine whether inclusion of sociodemographic information in foot and ankle RCTs depends on factors, such as specific journal, year of publication, and outcome study.

Of the 40 RCTs included in our query only a minority of studies reported data on race, ethnicity, insurance status, income, work status, or education. Race was the most commonly reported sociodemographic variable in the “Results” section (reported in 4/40 studies) and one of the most commonly reported sociodemographic factors in the other sections (reported in 6/40 studies). Income and work status, which were the other most commonly reported sociodemographic factors in the other sections, were only included in 6 studies. Similar to our findings, sociodemographic factors were often omitted from the majority of RCTs across surgical disciplines.^16^ When demographic information was included, race was one of the most frequently reported sociodemographic variables reported as well.^18^ Finally, inclusion of sociodemographic variables based on journal, year of publication, and outcome study was not statistically significant in our study, indicating that the lack of patient social and demographic data is pervasive across foot and ankle RCTs regardless of the type of study or journal publication, and that this has remained relatively unchanged over time.

Although patient’s social and demographic data are frequently omitted in foot and ankle RCTs, this information should not be overlooked. Many retrospective studies have described the impact of socioeconomic factors on both objective patient outcomes and subjective patient experience.^3,5-9,12,19-24^ Factors, such as race, ethnicity, and education level predicted outcomes for patients undergoing arthroplasty procedures in numerous studies.^5,22,23^ A literature review conducted by Pierce et al reported several studies that showed African American patients were twice as likely as Caucasian patients to experience an arterial injury during a total knee or total hip arthroplasty procedure. African American race was also associated with a statistically significant higher 30-day complication rate and readmission rate, a higher mortality rate, a higher rate of deep wound infections, and higher rate of undergoing a manipulation under anesthesia.^22^ Singh et al reported statistically significant differences in odds ratios for postoperative outcomes, such as duration of hospital stay, discharge to a rehabilitation facility, in-hospital transfusion, and in-hospital revision surgery associated with race in patients after total ankle arthroplasty.^12^ A study by Lavernia et al showed that African Americans presented with worse perceived pain and function both preoperatively and postoperatively following total hip and total knee arthroplasty compared with Caucasians.^6^ Bernstein et al found statistically significant differences in patient satisfaction and functional outcomes of foot and ankle patients in the highest income bracket compared with the lowest income bracket.^13^

Our study evaluates the incidence of sociodemographic reporting in RCTs; however, numerous studies have shown how sociodemographic patient factors impact patient treatment, patient outcomes, and overall patient experiences. Because of the clinical relevance of patients’ sociodemographic information, sociodemographic factors need to be considered in order to conduct high-quality research. The number of RCTs in foot and ankle surgery is low compared with other subspecialties in orthopaedics—thus, it is exceedingly important to include sociodemographic data in future studies.

Limitations to our study include the limited sample size of our study. We based our analysis from 40 RCTs published in 17 different journals over 6 years. Furthermore, we manually queried the studies included in our study. Although electronic databases would more accurately return a comprehensive search for foot and ankle RCTs, we opted to manually query the articles in our analysis in order to eliminate potential sources of bias.^25^ Despite our attempts to include a thorough analysis of foot and ankle RCTs, there may have been some studies that were omitted from our literature review.

In conclusion, our study demonstrates that there is a significant lack of sociodemographic data reported across foot and ankle RCTs over the past 6 years. The relationship between patients’ social, demographic, and economic characteristics and the impact on their experience, surgical outcomes, and postoperative complications is complex but critical to understand. Many studies have established these connections through retrospective data analyses in foot and ankle patients; however, investigation of these individual variables through RCTs is limited.^7^ The study design of RCTs is particularly amenable to intentionally recruiting and incorporating a diverse patient study population. Reporting sociodemographic factors in foot and ankle RCTs will not only facilitate applicability and generalizability across patient populations but also expose potential biases and increase comparability and validity of the results.^18^ Furthermore, incorporating patient demographics in the data analysis of foot and ankle RCTs provides opportunities to explore treatment disparities and unpack potential key relationships that affect patient outcomes from both the physician and patient perspective.^16^ Increasing evidence suggests that without considering the sociodemographic variables in a study, important clinical correlations may be missed.^7,18^ Here we reveal the lack of sociodemographic variables in RCT analyses and therefore recommend that journals establish more specific and detailed guidelines for reporting patients’ social, demographic, and economic information.
